# Correlations between Root-Associated Microorganisms and Peach Replant Disease Symptoms in a California Soil

**DOI:** 10.1371/journal.pone.0046420

**Published:** 2012-10-05

**Authors:** Jiue-in Yang, Paul M. Ruegger, Michael V. McKenry, J. Ole Becker, James Borneman

**Affiliations:** 1 Department of Nematology, University of California Riverside, Riverside, California, United States of America; 2 Department of Plant Pathology and Microbiology, University of California Riverside, Riverside, California, United States of America; University of Wisconsin-Milwaukee, United States of America

## Abstract

**Background:**

Replant disease often occurs when certain crops are “replanted” in a soil that had previously supported the same or similar plant species. This disease typically leads to reductions in plant growth, crop yields, and production duration, and its etiology remains ill-defined. The objective of this study was to identify microorganisms associated with peach replant disease symptoms at a field location in California, USA. Soil samples were subjected to treatments to create various levels of replant disease symptoms. Clonal peach seedlings were grown in the treated soils in greenhouse trials. After 6 weeks, plant growth parameters were measured, and both culture and culture-independent analyses were performed to identify root-associated bacteria, fungi and stramenopiles.

**Results:**

A total of 295,785 bacterial operational taxonomic units (OTU) were identified by an Illumina-based, high throughput sequence analysis of rRNA genes. Among the 60 most abundant OTUs, 27 showed significant (*P*<0.05) negative correlation with peach shoot weights while 10 were positively correlated. Most of these OTUs belonged to the bacterial phylum Proteobacteria (96%), including the classes Gammaproteobacteria (44.4%), Betaproteobacteria (33.3%) and Alphaproteobacteria (22.2%), and the orders Pseudomonadales, Burkholderiales, Chromatiales, Rhodocyclales, and Sphingomonadales. The most abundant fungi were *Trichoderma asperellum*, *Trichoderma virens, Fusarium oxysporum*, *Ceratocystis fimbriata* and *Fusarium solani*. The most abundant stramenopiles were *Pythium vexans*, *Pythium violae* and an unidentified *Aplanochytrium* species. Validation experiments using sequence-selective quantitative PCR analyses identified negative and positive associations between *P. vexans* and *Trichoderma* spp. and peach shoot weights, respectively.

**Conclusions:**

This study identified numerous microorganisms associated with peach replant symptoms, some of which have been previously identified while others represent new candidates. Subsequent Koch's postulates investigations will assess their possible roles in this replant disease.

## Introduction

Replant diseases of pome, stone fruits, and closely related ornamentals have been reported in all of the major crop-growing regions of the world [1]–[3]. The problems are typically expressed as a dramatic reduction in plant growth and vigor, with a subsequent reduction in yield and a shortened production life. Annual losses in California alone are estimated to be 10–20% [4], [5]. As the causes of replant diseases are complex, may differ among regions, and are frequently ill-defined, management strategies have until recently relied on broad-spectrum fumigants such as methyl bromide. The world-wide phase-out of this fumigant, and the lack of available alternatives with similar efficacy, creates an urgent need for identifying the organism(s) that cause replant disease – a discovery that would enable the development of targeted management strategies, and therefore reduce reliance on such fumigants.

Numerous factors have been implicated in replant disease etiology. Putative causal agents have varied considerably among geographic regions and among orchards in the same region. Abiotic factors such as nutrition, soil structure, and phytotoxic metabolites from roots of previous crops have been implicated [6]–[9]. Microorganisms including a variety of bacteria, fungi, and oomycetes have also been implicated [10]–[30]. For example, replant disease in *Prunus* has been associated with an increase in rhizosphere bacilli and higher populations of cyanogenic microorganisms [10].

In this study, we examined a peach replant disease soil located in the center of California's San Joachim Valley near Parlier, CA, USA. We first determined that there was a biological component to the replant disease symptoms. We then used a population-based approach to identify root-associated bacteria, fungi, and stramenopiles that correlated with replant disease symptoms. Finally, sequence-selective qPCR assays were used to validate selected associations.

## Results

### Replant Disease Soil

A series of investigations were performed on a soil exhibiting replant disease properties located in Parlier, California, USA. When peach seedlings were planted in this field, they showed reductions in height and trunk-width 10 weeks after planting when compared to seedlings grown in soil fumigated with 1,3-dichloropropene (Telone II®) at 332 lb/acre, broadcast. In addition, the root systems had less developed feeder roots and were slightly darker in color than the ones in the fumigated soil [31].

To further examine the nature of this phenomenon, we performed greenhouse trials comparing plant growth parameters of peach seedlings grown in autoclaved and non-autoclaved portions of this soil. After 10 weeks, root weights, shoot weights, and shoot length were measured. In all cases, plant growth was better in the autoclaved portions (Table 1), indicating a biological component was contributing to the replant disease symptoms.

**Table 1:** Plant growth parameters of peach seedlings grown in autoclaved and non-autoclaved portions of a replant soil for 10 weeks in greenhouse experiments.

Soil type	Plant growth parameters^a^				
	Fresh weight (g)		Dry weight (g)		Length (cm)
	Roots	Shoots	Roots	Shoots	Shoots
Replant soil	11.17±3.79 x	12.33±5.20 x	2.82±0.84 x	4.36±1.82 x	98.56±20.87 x
Autoclaved replant soil	15.69±3.05 y	22.41±3.64 y	3.61±0.81 y	8.28±1.52 y	126.38±7.73 y

a Values and standard deviations are the means of 8 replicates pots. Values in columns followed by the same letter are not significantly (*P*<0.05) different.

### Bacterial Associations

**Figure 1:**
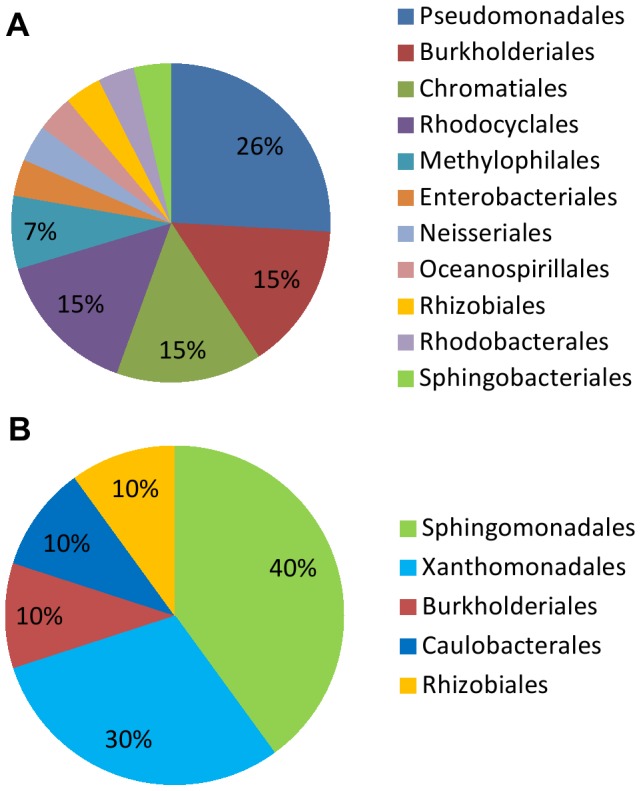
Relative abundance of the most prevalent bacterial orders associated (*P*<0.05) with fresh peach shoot weights. A. Negative associations. B. Positive associations.

An Illumina-based, high throughput sequencing analysis of the small-subunit rRNA gene was used to examine root-associated bacteria from plants exhibiting different levels of replant disease symptoms. A total of 295,785 bacterial operational taxonomic units (OTU) were identified, 5,629 of which had significant correlation (*P*<0.05) with peach shoot weights. We posit that microbes are more likely to impact the host plant if their population densities are high. Thus, our subsequent analyses focused on the most abundant (>10,000 reads) OTUs, 27 of which showed significant (*P*<0.05) negative correlation with peach shoot weights (Table 2) while 10 were positively correlated (Table 3). Most of the 27 negatively associated OTUs belonged to the bacterial phylum Proteobacteria (96%), including the classes Gammaproteobacteria (44.4%), Betaproteobacteria (33.3%) and Alphaproteobacteria (22.2%). The predominant orders from these 37 OTUs included Pseudomonadales, Burkholderiales, Chromatiales, Rhodocyclales, and Sphingomonadales (Figure 1).

**Table 2:** Most abundant bacterial OTUs negatively associated with fresh peach shoot weights.

OTU designation	Nearest cultured relative (accession) (% identity)^a^	Nearest uncultured relative accession (% identity)^a^	Abundance (% of total reads)	Correlation coefficient (r^b^)	Probability (*P*)
278666	*Hydrogenophaga flava* (AB681848) (98%)	HQ120802 (98%)	0.094	−0.687	0.000
243054	*Aquabacterium* sp. (FN692032) (98%)	HE583131 (98%)	0.082	−0.686	0.000
61	*Cupriavidus* sp. (AB681843) (100%)	HQ783640 (100%)	0.042	−0.626	0.000
35800	*Pseudogulbenkiania* sp. (AP012224) (98%)	AB657767 (98%)	0.059	−0.611	0.000
26781	*Pseudomonas pachastrellae* (HQ425676) (94%)	FJ568592 (100%)	0.198	−0.567	0.000
129755	Bacterium MI-37 (AB529705) (95%)	FJ568592 (97%)	0.065	−0.561	0.000
172482	*Azoarcus* sp. (AP012304) (100%)	JN825463 (100%)	0.088	−0.554	0.001
234080	*Azoarcus* sp. (AP012304) (96%)	JN825463 (96%)	0.032	−0.551	0.001
250441	*Thiocystis violacea* (FN293059) (95%)	JF990363 (98%)	0.111	−0.530	0.002
115618	*Pseudomonas fluorescens* (JN411289) (98%)	AB579016 (98%)	0.128	−0.520	0.002
273727	*Pseudomonas putida* (JN411453) (96%)	AB579016 (96%)	0.037	−0.503	0.003
210082	*Dechloromonas* sp. (GU202936) (100%)	GU179639 (100%)	0.044	−0.493	0.004
193280	*Pseudomonas* sp. (HE586886) (100%)	JQ032435 (100%)	0.023	−0.484	0.005
236351	*Rahnella aquatilis* (JQ014185) (100%)	JN998890 (100%)	0.036	−0.481	0.005
288392	*Ramlibacter* sp. (HQ323427) (98%)	FQ690103 (98%)	0.181	−0.468	0.007
184527	*Rhizobacter* sp. (HE616175) (100%)	FQ659876 (100%)	0.065	−0.458	0.008
244218	*Methylophaga thalassica* (AB681780) (95%)	HQ697540 (100%)	0.041	−0.427	0.015
207860	*Pseudomonas taiwanensis* (JQ014182) (100%)	HE650703 (100%)	0.059	−0.415	0.018
273656	*Methylobacillus* sp. (EU194898) (97%)	FQ659555 (98%)	0.155	−0.405	0.022
17162	*Bradyrhizobium* sp. (HQ836187) (98%)	JN540015 (98%)	0.259	−0.387	0.029
167695	*Methylophilus leisingeri* (NR_041258) (100%)	AB635923 (100%)	0.071	−0.387	0.029
246943	*Pseudomonas* sp. (FN995250) (94%)	FQ659619 (97%)	0.536	−0.374	0.035
166091	*Woodsholea maritima* (FM886859) (97%)	HE614733 (99%)	0.062	−0.368	0.038
234039	*Terrimonas lutea* (NR_041250) (100%)	FQ706675 (100%)	0.060	−0.367	0.040
11757	*Thiocystis violacea* (FN293059) (97%)	FR853185 (99%)	0.046	−0.361	0.042
164910	*Pseudomonas* sp. (FN995250) (92%)	FQ659619 (95%)	0.110	−0.361	0.042
32731	*Cellvibrio japonicus* (CP000934) (99%)	HQ691969 (98%)	0.119	−0.360	0.043

a % identity values are from analyses using BLAST (NCBI) where coverage was at least 96%.

b r is the Pearson's correlation coefficient.

**Table 3:** Most abundant bacterial OTUs positively associated with fresh peach shoot weights.

OTU designation	Nearest cultured relative (accession) (% identity)^a^	Nearest uncultured relative accession (% identity)^a^	Abundance (% of total reads)	Correlation coefficient (*r* b)	Probability (*P*)
164017	*Massilia aerilata* (HQ406763) (98%)	JN590660 (98%)	0.202	0.546	0.012
101298	*Thermomonas haemolytica* (GU195191) (98%)	FQ680347 (98%)	0.339	0.532	0.017
286079	*Sphingopyxis* sp. (JF297627) (99%)	HQ118566 (99%)	0.132	0.447	0.010
66648	*Novosphingobium naphthalenivorans* (AB681685) (99%)	HQ754243 (99%)	0.154	0.442	0.011
173712	*Novosphingobium naphthalenivorans* (AB681685) (98%)	HQ754243 (98%)	0.647	0.431	0.014
259461	*Sphingopyxis* sp. (JF297627) (98%)	HQ118566 (98%)	0.427	0.423	0.016
233081	*Rhodopseudomonas palustris* (AB689796) (98%)	JN863157 (98%)	0.140	0.422	0.016
162892	*Novosphingobium subterraneum* (HM032869) (98%)	FQ741870 (98%)	0.464	0.398	0.024
275502	*Dyella* sp. (GQ369135) (100%)	JF341880 (100%)	0.259	0.394	0.025
30925	*Rhodanobacter lindaniclasticus* (L76222) (100%)	JF341837 (100%)	0.493	0.366	0.039

a % identity values are from analyses using BLAST (NCBI) where coverage was at least 96%.

b r is the Pearson's correlation coefficient.

### Fungal and Stramenopile Associations

To identify fungi and stramenopiles associated with the replant disease symptoms, both culture and culture-independent analyses were performed on root-associated organisms from plants exhibiting different levels of replant disease symptoms. For the culture-based studies, 295 fungal and stramenopile isolates were obtained and identified by sequence analysis of the rRNA internal transcribed spacer (ITS). For the culture-independent analysis, 274 small-subunit rRNA gene clones were analyzed. In both the fungal and stramenopile analyses, as expected, there was a considerably greater number of phylotypes detected in the culture-independent analysis compared to the culture-based analysis.

**Figure 2:**
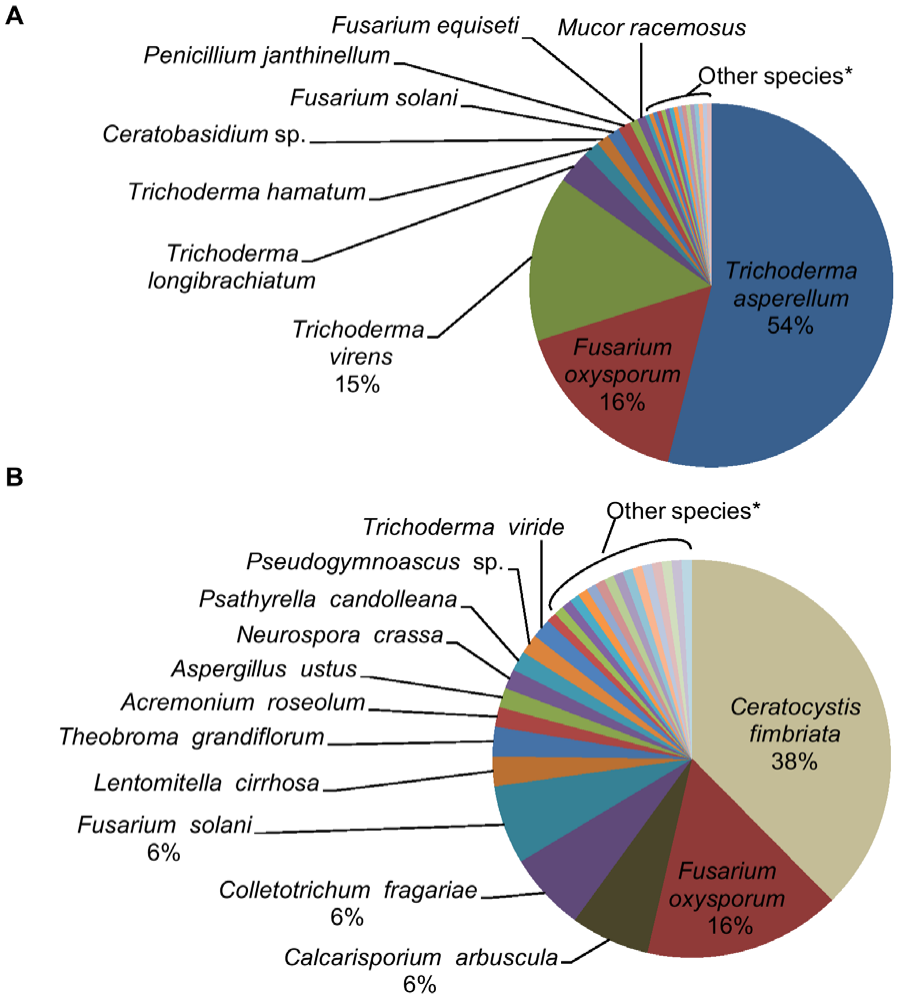
Relative abundance of fungi from peach seedling roots grown in a soil exhibiting peach replant disease symptoms. A. Cultured-based analysis; values are % of 269 isolates. *Species less than 1% included: *Apodus oryzae*, *Ceratocystis fimbriata*, *Clonostachys divergens*, *Cunninghamella echinulata*, *Gelasinospora brevispora*, *Hypocrea koningii*, *Hypocrea sp.*, *Mortierella alpina*, *Mortierella elongata*, *Mortierella minutissima*, *Penicillium simplicissimum*, *Sclerostagonospora opuntiae*, *Sporormia subticinensis*, *Trichoderma gamsii*, *Trichoderma pubescens*, and *Trichoderma viride*. B. Culture-independent analysis; values are % of 130 sequences. *Species less than 1% included: *Acremonium sclerotigenum*, *Aspergillus niger*, *Brachyconidiellopsis sp.*, *Craterocolla cerasi*, *Elaphocordyceps ophioglossoides*, *Engyodontium album*, *Halophytophthora vesicular*, *Hypomyces chrysospermus*, *Lithothelium septemseptatum*, *Metarhiziopsis microspore*, *Neokarlingia chitinophila*, *Nomuraea rileyi*, *Ophiocordyceps konnoana*, *Sebacina vermifera*, *Verticillium dahlia*, and *Volutella ciliata*. For A and B, taxa without numbers have relative abundance values between 1% and 5%.

**Figure 3:**
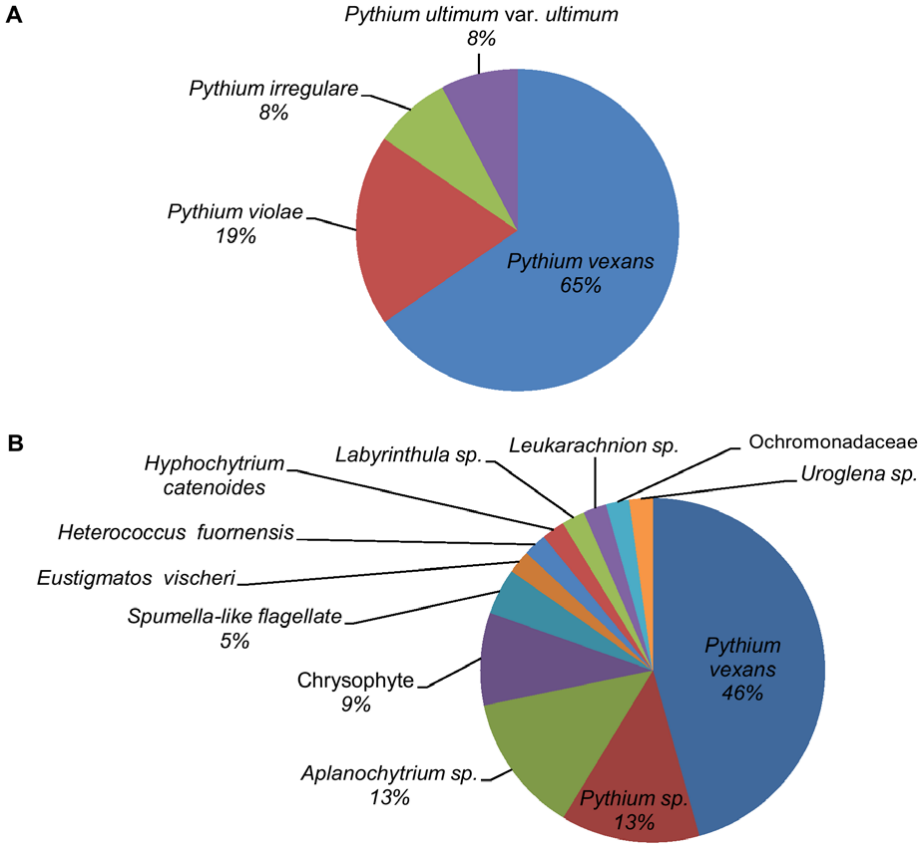
Relative abundance of stramenopiles from peach seedling roots grown in soil exhibiting peach replant disease symptoms. A. Cultured isolates; values are % of 26 isolates. B. Culture-independent analysis; values are % of 48 sequences. Taxa without numbers have relative abundance values of less than 5%.

The most abundant fungi isolated from roots grown in the replant soil were *Trichoderma asperellum* (54%), *Fusarium oxysporum* (16%), and *Trichoderma virens* (15%) (Figure 2A). In contrast, the most abundant fungi obtained from the culture-independent analysis of roots grown in the replant soil were *Ceratocystis fimbriata* (38%) and *F. oxysporum* (16%) (Figure 2B). The most abundant stramenopiles isolated from roots grown in the replant soil were the oomycetes *Pythium vexans* (65%), *Pythium violae* (19%), and *Pythium irregulare* (8%) (Figure 3A). The most abundant stramenopiles obtained from the culture-independent analysis of roots grown in the replant soil were *Pythium vexans* (46%), an unidentified *Pythium* species (13%), and an unidentified *Aplanochytrium* species (13%) (Figure 3B).

The most abundant fungal and stramenopile isolates and phylotypes were subjected to further analysis using sequence-selective qPCR assays targeting the ITS region. For the phylotypes identified by the small-subunit rRNA gene analyses, chromosomal walking procedures were used to obtain the ITS sequences. Using an assay targeting both *Trichoderma* species, a positive association was detected with plant shoot weights [log_10_ rRNA copy number = 5.97+0.0648 (grams of shoots); *P* = 0.012; R^2^ = 6.5%, n = 116]. Using an assay targeting *P. vexans*, a negative association was detected with plant shoot weights [log_10_ rRNA copy number = 5.01−0.0931 (grams of shoots); *P* = 0.008; R^2^ = 8.7%, n = 116]. Using an assay targeting *C. fimbriata*, a negative association was detected with plant shoot weights [log10 rRNA copy number = 7.11−0.0574 (grams of shoots); P = 0.097; R^2^ = 2.7%, n = 104].

## Discussion

To our knowledge, this study presents the first use of high throughput sequencing to examine bacteria associated with replant disease. Given the large number of OTUs identified, it was not surprising that we identified considerable numbers of organisms correlating with plant growth parameters, most of which have not been previously associated with replant disease. In addition, most of these bacteria are not known to be plant pathogens or have functions that could be readily attributed to replant disease properties. Ultimately, determining the roles that these bacteria may play in replant disease will require additional experimentation, including fulfilling Koch's postulates.

**Figure 4:**
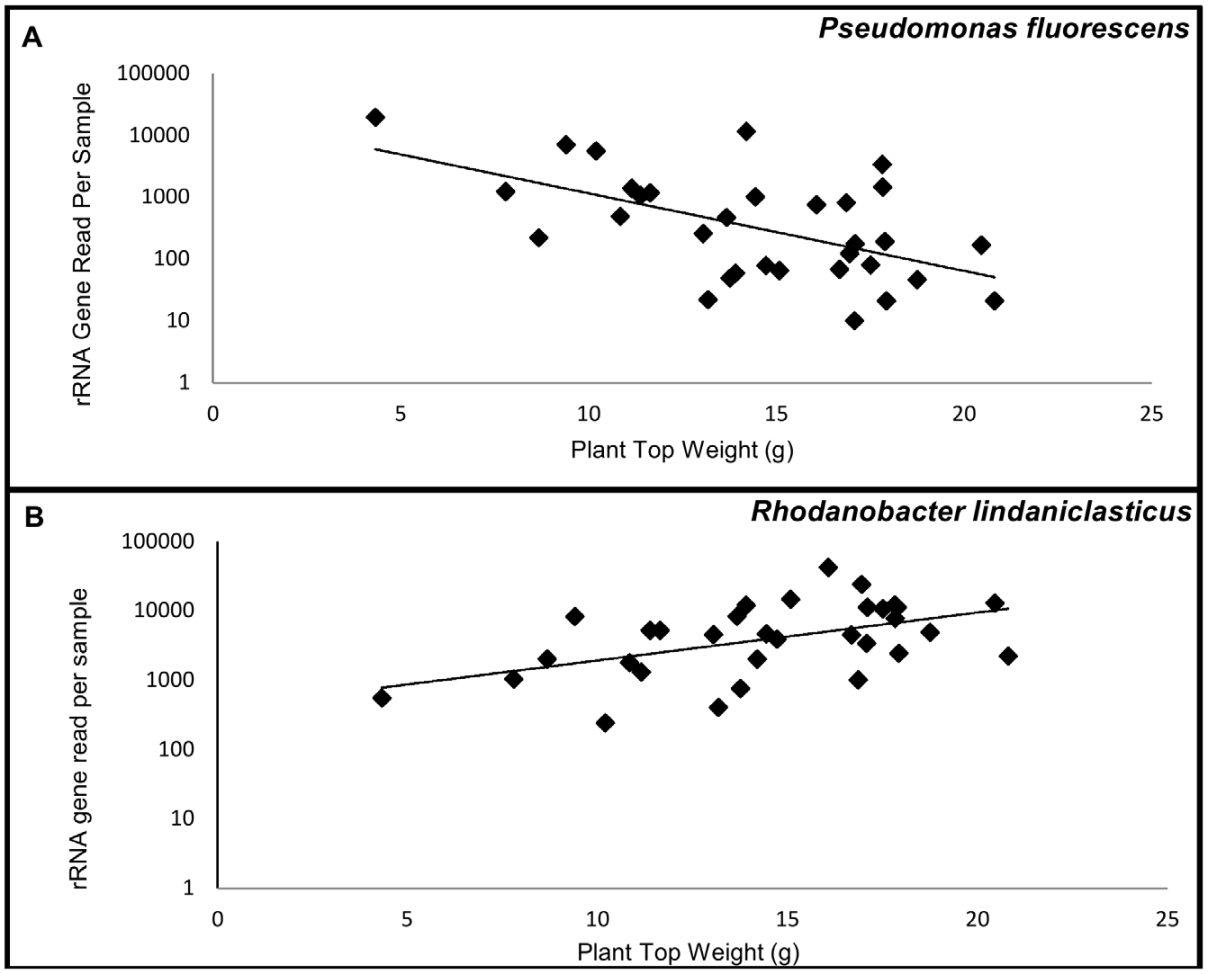
Relationships between bacteria and fresh peach shoot weights. A. *Pseudomonas fluorescens*. B. *Rhodanobacter lindaniclasticus*. Regression equations are (A) [log_10_ reads per sample = 4.02−0.146 (grams of shoots); *P* = 0.015; R^2^ = 18.3%, n = 32] and (B) [log_10_ reads per sample = 2.59+0.069 (grams of shoots); *P* = 0.004; R^2^ = 25.0%, n = 32]. Lines are from regression analyses.

Findings from this study that are consistent with prior work include the identification of several OTUs from the genus *Pseudomonas*, which exhibited negative correlations with peach shoot weights. These phylotypes possess rRNA genes with high sequence identities to *P. pachastrellae*, *P. putida*, *P. fluorescens*, *P. straminea*, *P. fulva*, *P. taiwanensis*, and *P. monteilii* (Table 2, and example in Figure 4A). In prior investigations of root-associated bacteria and replant disease, pseudomonads were shown to be more abundant in the rhizoplane of plants grown in grapevine replant soils [32].

Bacteria from several taxonomic groups, including *Pseudomonas*, have been proposed to play a role in replant disease etiology through the production of hydrogen cyanide (HCN). When soils were amended with peach roots dried at 80°C, they caused retarded growth of peach seedlings [6]. These investigators also showed that peach roots contained considerable amounts of an HCN precursor, and roots grown in the replant soils harbored heat-resistant bacilli able to transform this precursor into HCN. While our investigation did not detect bacilli that negatively correlated with plant weights, we did identify significant negative associations with pseudomonas (Table 2), which are also known to produce HCN [33], [34]. Other investigations have reported related findings including higher levels of HCN-producing bacilli in the rhizosphere of peach plants grown in a replant soil [10]. In addition, HCN-producing *Pseudomonas* species were isolated from the rhizosphere in an apple replant site [35]. Variation among the results from the aforementioned studies could involve differences in the subtypes of replant diseases and/or the methods used to identify the microbial communities.

Another putatively causal taxon is the actinobacteria, formerly called actinomycetes. Prior microscopic investigations identified large numbers of actinobacteria growing in the epidermal and cortical tissue of apple seedling rootlets grown in replant disease soils, and very few actinobacteria in rootlets grown in non-replant soils [36], [37]. In addition, the amount of plant tissue damage was proportional to the number of actinobacteria [36]. In our study, we also identified several actinobacterial OTUs that were negatively associated with plant shoot weights, although they were not present in high numbers (Table 4).

**Table 4:** Actinobacterial OTUs negatively associated with fresh peach shoot weights.

OTU designation	Nearest cultured relative (accession) (% identity)^a^	Nearest uncultured relative accession (% identity)^a^	Abundance (% of total reads)	Correlation coefficient (*r* b)	Probability (*P*)
76253	*Catellatospora* sp. (KC-EP-S6) (FJ711222) (100%)	JF982278 (100%)	<0.001	−0.826	0.000
22707	*Solirubrobacter* sp. L64 (FJ459990) (90%)	JF987729 (100%)	<0.001	−0.774	0.004
105414	*Gaiella occulta* (JF423906) (92%)	HM187195 (98%)	<0.001	−0.744	0.025
66985	*Aciditerrimonas ferrireducens* (AB517669) (84%)	AJ616079 (97%)	<0.001	−0.740	0.027
101423	*Phycicoccus* sp. P3703 (JQ419657) (98%)	CU919577 (99%)	<0.001	−0.738	0.031
163353	*Virgisporangium ochraceum* (AB546280) (97%)	FJ479084 (100%)	<0.001	−0.734	0.037
266579	*Gaiella occulta* (JF423906) (94%)	AB656050 (100%)	0.001	−0.732	0.042

a % identity values are from analyses using BLAST (NCBI) where coverage was at least 88%.

b r is the Pearson's correlation coefficient; probability values were adjusted using the Bonferroni correction method.

It was surprising to find that Xanthomonadaceae was one of the dominant taxa exhibiting a positive association with plant shoot weights (Table 3, and example in Figure 4B), because these bacteria, and specifically pathovars in the order Xanthomonas, have been shown to cause diseases on at least 124 monocotyledons and 268 dicotyledons [38]. Our results suggest a possible beneficial role of this bacterial group, where these organisms may interact with plants and/or other microbes, leading to plant growth promotion.

Numerous oomycetes also have been implicated in replant disease etiology [12], [13], [16], [18], [20], [22]–[24], [26]–[28]. In our study, *P. vexans* was frequently detected by both culture-based and culture-independent analyses. In addition, our sequence-selective qPCR analysis showed a negative correlation between *P. vexans* rRNA gene numbers and peach shoot weights. However, in another peach replant study, *P. vexans* was not significantly correlated with plant biomass [39]. Such site dependent variation has been observed in replant diseases of other crops. For example, *P. vexans* was shown to be pathogenic to apple seedlings in one study [24] yet exhibited biological control efficacy in another [20]. This phenomenon could be due to virulence differences among the *P. vexans* strains and/or varying interactions with abiotic or biotic features of the soils [40], [41].

Several features of *P. vexans* could allow it to contribute to replant disease symptoms. Zoospores of *P. vexans* are attracted to roots at zones of elongation and at breaks in cortical tissue associated with lateral root emersion [42]. In addition, two elicitin-like proteins (Vex1 and Vex2) secreted by *P. vexans* appear to induce a necrotic and hypersensitive response in a manner similar to that observed in *Phytophthora* species [43].

Many fungi also have been implicated in peach replant diseases. For example, *Fusarium equiseti*, *Fusarium moniliforme*, *Fusarium oxysporum*, *Fusarium solani*, *Alternaria tenuis*, *Myrothecium verrucaria*, and *Mycelia sterilia* were frequently isolated from the rhizosphere or roots of peach trees grown in a replant soil and found to be parasitic to the roots [29]. In Kent, England, *Thielaviopsis basicola* was implicated in cherry and plum replant disease [15], [25], [30]. *Armillaria* and *Verticillium* were associated with peach and almond replant symptoms in California [30]. In Italy, several species of *Fusarium*, *Penicillium*, *Aspergillus*, and *Trichoderma* were common isolates from peach replant soils [17].

Several of the most abundant fungi identified in our study have been previously implicated in replant disease including the abovementioned *F. oxysporum* and *Trichoderma* spp. In addition, the most abundant fungus in our replant soil identified by the culture-independent analysis was *Ceratocystis fimbriata*. Although *C. fimbriata* has been described as the pathogen of mallet wound canker on almond, peach, apricot, and coffee [44], [45], and it is the causal agent of many other wilt and rot diseases of sweet potato, poplar, cocoa, citrus, gmelina, and *Eucalyptus* species [46]–[48], to our knowledge it has not been previously associated with peach replant disease.

Our investigations also identified two *Trichoderma* species that were frequently isolated from peach roots, and that exhibited a positive association with plant shoot weights via a sequence-selective qPCR analysis. These results suggest that these fungi may be inhibiting the causal microbes and/or acting as a plant growth promoter. *Trichoderma* species can inhabit a variety of soil and plant niches [49]. The genus contains members that are plant growth promoters that act through a variety of mechanisms. *Trichoderma* species produce several lytic enzymes and antibiotics against plant pathogens, and several products made from these fungi have been commercially marketed as biopesticides, biofertilizers, and soil amendments [50]. *Trichoderma virens* is one of the well-studied species, which exhibits mycoparasitic characteristics and the ability to produce several potent epithiodiketopiperazine antibiotics that inhibit oomycetes such as *Pythium* and *Phytophthora* species. It also produces a mixture of peptaibols, which are linear peptide antibiotics that might affect certain bacteria and fungi [51]. Some strains of *T. asperellum* and *T. harzianum* are capable of activating plant defense responses [52], [53]. Strains of *T. asperellum* also have been shown to suppress important plant pathogens including *Phytophthora megakarya* [54], *Fusarium oxysporum* f. sp. *lycopersici* [55], *Rhizoctonia solani* [56], and *Meloidogyne javanica* [57].

In sum, this study identified large numbers of microorganisms associated with peach replant disease symptoms in a Californian soil. Such associations point toward organisms that could be causing or inhibiting the replant disease, or that are simply responding to changes in the environment caused by the disease. Future investigations that assess cause and effect, such as Koch's postulates experimentation, will be needed to further define the roles of these organisms.

## Materials and Methods

### Greenhouse Trials

Greenhouse trials were performed to (i) determine if there was a biological component causing the replant disease symptoms and to (ii) create soils with various levels of the replant disease symptoms for microbial community analyses. Soil (upper 30-cm) was collected from a field at the University of California Kearney Agricultural Research and Extension Center in Parlier, California, USA, where replant disease symptoms were observed on Nemaguard rootstocks 10 weeks after planting [31]. Soil texture parameters were 62% sand, 30% silt, and 8% clay. Soil was passed through a metal sieve with 12-mm openings prior to use in greenhouse experiments.

To determine if there was a biological component causing the replant disease symptoms, peach seedling clones were planted in the untreated replant soil and replant soil that had been autoclaved for 2 hours at 121°C. Plastic pots with drain holes were double-cupped and filled with 800-cm^3^ of the two soils. Each pot was planted with one two-month-old Nemaguard peach seedling clone, donated by Duarte Nursery, Hughson, CA. Each pot was fertilized with 7-g of slow-release fertilizer (Sierra 17-6-10 plus Minors, Scotts-Sierra Horticultural Products Company, Marysville, OH) and watered as needed. Trials were performed in a greenhouse, arranged in a randomized complete design with 8 replicates for each soil treatment. After 10 weeks, root weights, shoot weights and shoot length were measured.

To create soils with various levels of the replant disease symptoms for the microbial community analyses, soils were (i) temperature-treated or (ii) diluted with various amounts of autoclaved soil. For the temperature treatments, soils were exposed to room temperature, 40°C, 50°C, 60°C and 70°C. Soil samples (∼1 kg) were double-bagged and submerged in a water bath, and held for 30 minutes once the center of the sample reached the target temperature. The bags were then cooled to room temperature under running tap water. All samples of the same treatment were pooled and mixed. For the dilution treatments, soils were mixed with different percentages of autoclaved soil (121°C for 2 hours) at ratios of non-treated to autoclaved soil of 100∶0, 10∶90, 1∶99, 0.1∶99.9 and 0∶100. Treated soils were aerated at room temperature for 2 days prior to use. As described above, pots were filled with soil, planted with peach seedling clones, fertilized and watered. Trials were performed in a greenhouse, arranged in a randomized complete design with six replicates for each soil treatment, and performed twice. After 6 weeks, plant shoots were cut off 10-cm above the soil level and weighed. Shoot lengths of each branch were measured from the main stem. Plant dry weights were measured after 3-days in a drying oven at 125°C. Root tip samples (200 mg) from each plant were collected and stored at −20°C for DNA extraction. Fine root tips were collected and stored in sterile tubes at room temperature for culturing of fungi and oomycetes. Plant growth parameters obtained from the greenhouse trials were subjected to ANOVA and two-tailed student t-tests using Microsoft Excel 2007 (Microsoft, Redmond, WA).

### Isolation of Fungi and Stramenopiles

Pieces of fine root tips from each of the non-treated replant soils were collected at the end of the trials, stored at room temperature and processed for culturing within 24 hours after sampling. From each replicate pot, 12 pieces of 3-cm-long root tips were rinsed with ultrapure water for 15 seconds, dried by pressing between paper towels, placed on 1% water agar, and incubated at room temperature. Fungi and oomycetes that emerged from the root surfaces during the first 36 hours were sub-cultured on new 1% water agar plates. The hyphal-tip method was used to obtain pure cultures. Isolates were identified by rRNA gene sequence analysis (described below).

### DNA Extraction

DNA was extracted from root tip samples collected at the end of the greenhouse trials and fungi and oomycetes cultured from the roots. Two hundred milligrams of root tips or fungal hyphae were used for each DNA extraction. Genomic DNA was extracted using the FastDNA Spin Kit for Soil (Qbiogene, Carlsbad, CA) as described by the manufacturer using a 90 second bead-beating step in a FastPrep Instrument (Qbiogene) and a 5.5 setting. The extraction product was further purified by electrophoresis in 1% agarose gels. DNA larger than 3 Kb was isolated by using a MinElute Gel Extraction Kit (Qiagen, Valencia, CA), without use of UV lighter or ethidium bromide.

### Bacterial rRNA Gene Sequencing

A high throughput sequencing analysis of the bacterial small-subunit rRNA genes was performed using genomic DNA extracted from the root samples collected at the end of the greenhouse trials as templates. Root samples used were from seedlings grown in the non-treated to autoclaved soil ratios of 100∶0, 10∶90, 1∶99, and the temperature treatments of 40°C and 50°C. One hundred microliter amplification reactions were performed in an MJ Research PTC-200 thermal cycler (Bio-Rad Inc., Hercules, CA) and contained: 50 mM Tris (pH 8.3), 500 µg/ml bovine serum albumin (BSA), 2.5 mM MgCl_2_, 250 µM of each deoxynucleotide triphosphate (dNTP), 400 nM of each primer, 4 µl of DNA template, and 2.5 units JumpStart *Taq* DNA polymerase (Sigma-Aldrich, St. Louis, MO). The PCR primers (F515/R806) targeted a portion of the 16S rRNA gene containing the hypervariable V4 region, with the reverse primers including a 12-bp barcode (Tables S1 and S2) [58]. Thermal cycling parameters were 94°C for 5 minutes; 35 cycles of 94°C for 20 seconds, 50°C for 20 seconds, and 72°C for 30 seconds, and followed by 72°C for 10 minutes. PCR products were purified using a MinElute Gel Extraction Kit (Qiagen) except PB buffer was substituted with QG buffer. PCR products were diluted to 20 ng/µl. DNA sequencing was performed using an Illumina HiSeq 2000 (Illumina, Inc., San Diego, CA). One hundred base sequencing reads of the 5′ end of the amplicons and seven base barcode reads were obtained using the sequencing primers listed in Table S3. De-multiplexing, quality control and OTU binning were performed using QIIME [59]. The 5′ base of the barcodes was not used for de-multiplexing because of low quality reads at this position; however, all 32 codes (one for each of the 32 samples) were distinguishable using the last six bases. Low quality sequences were removed using the following parameters: Q20, minimum number of consecutive high quality base calls = 100 bp, maximum number of N characters allowed = 4, maximum number of consecutive low quality base calls allowed before truncating a read = 4. The total number of sequencing reads was 71,320,513. Numbers of sequences removed using the aforementioned quality control parameters were: barcode errors (3,893,840), reads too short in length (17,427,455), and too many Ns (4,072,554). OTUs were binned at 97% identity.

### PCR Amplification of Stramenopile and Fungal rRNA Genes

For the culture-independent analysis, root DNA extracted from plants exhibiting a wide range of replant disease symptoms from greenhouse trials were used as templates. Ten microliter amplification reactions were performed in 10-µl glass capillary tubes using a RapidCycler (Idaho Technologies, Salt Lake City, UT) containing the following reagents: 50 mM Tris (pH 8.3), 500 mg/ml bovine serum albumin (BSA), 2.5 mM MgCl_2_, 250 mM of each dNTP, 400 nM of each forward and reverse primer, 1-µl (∼66 ng) of peach root DNA and 0.5 units *Taq* DNA polymerase. Fungi-selective primers were nu-SSU-0817-5 (TTAGCATGGAATAATRRAATAGGA) and nu-SSU-1536-3 (ATTGCAATGCYCTATCCCCA) [60] while stramenopiles were StramenoSSUF1 (GATGATTAGATACCATCGTA) and StramenoSSUR2 (AAAGGGCAGGGACGT) [39], with PCR products being ∼762 bp and ∼638 bp, respectively. Thermal cycling parameters were 94°C for 5 minutes; 35 cycles of 94°C for 20 seconds, X°C for 30 seconds and 72°C for 40 seconds; followed by 72°C for 5 minutes, where X = 55 for fungi and 59 for stramenopiles.

For the culture-based analyses, fungal and stramenopile (which were all oomycetes) isolates were identified by rRNA gene analyses. rRNA gene primers ITS1FUSER (GGGAAAGUCTTGGTCATTTAGAGGAAGTAA) [61] and ITS4USER (TCCTCCGCTTATTGATATGC) [62] were used with the following thermal cycling conditions: 94°C for 5 minutes, followed by 40 cycles of 94°C for 20 seconds, 52°C for 20 seconds, 72°C for 40 seconds, and a final incubation at 72°C for 5 minutes. PCR master mixes were prepared as described above in this subsection.

To obtain the sequences of the internal transcribed spacer (ITS) region for *P. vexans* and *C. fimbriata* for the quantitative PCR assays, chromosome walking was conducted as follows. PCR was performed on peach root DNA using the following forward primers combined with ITS4 (TCCTCCGCTTATTGATATGC) [62]: PvexansSSUF3 (GGGACTTTTGGGTAATC) or CfimbSSUF1 (AGGTCCAGACACAG). The forward primers were designed using PRISE [63]. The thermal cycling conditions were 94°C for 5 minutes; 40 cycles of 94°C for 20 seconds, 52°C for 30 seconds and 72°C for 90 seconds; followed by 72°C for 10 minutes. Amplification products were gel isolated and cloned as described previously [39], and the nucleotide sequences were obtained as described below.

Nucleotide sequences of fungi and stramenopile rRNA gene fragments were determined using the ABI BigDye Terminator v3.1 Cycle Sequencing Kit and an ABI 3730xl DNA Analyzer (Applied Biosystems, Foster City, CA). Sequence identities were determined by an analyses using BLAST (NCBI) [64].

### Quantitative PCR

Real-time qPCR assays targeting *P. vexans*, *C. fimbriata*, and *Trichoderma* (targeting species *T. asperellum* and *T. virens*) were performed using a Bio-Rad iCycler MyiQTM Real-Time Detection System (Bio-Rad Laboratories, Inc). The templates were genomic DNA extracted from the root samples collected at the end of the greenhouse trials. Sequence-selective primers developed in this study were designed using PRISE software [63]. The selective primers for *P. vexans* were VexansITSF31 (GCTGCTGGCGCTTGAT) and VexansITSR31 (TTCGTCCCCACAGTATACTT). The primers for *C. fimbriata* were CfimbITSF2 (TCTTCCTTGACAGAGATG) and CfimbITSR9 (TCACTGAGCCATCCAA). The primers for *Trichoderma* species were TricoITSF9 (TCCGAGCGTCATTTCAA) and TricoITSR3 (GTGCAAACTACTGCGC). The targets were fragments of the ITS rRNA gene with sizes of 131-bp, 181-bp, and 126-bp, respectively. The thermal cycling conditions were 94°C for 5 minutes; X cycles of 94°C for 20 seconds, Y°C for 30 seconds and 72°C for Z seconds; followed by 72°C for 10 minutes; where (X, Y, Z) = (44, 69.5, 40) for *P. vexans*, (38, 66.5, 30) for *C. fimbriata*, and (42, 65, 30) for *Trichoderma*. The amplification reactions were performed in iCycler iQ PCR Plates with Optical Flat 8-Cap Strips (Bio-Rad Laboratories, Inc.). PCR amplifications were performed in 25-µl reactions contained the following reagents: 50 mM Tris (pH 8.3), 500 ug/ml bovine serum albumin (BSA), 2.5 mM MgCl_2_, 250 mM of each dNTP, 400 nM of each primer, 1-µl of template DNA (∼176 ng), 2-µl of 10X SYBR Green I (Invitrogen, Carlsbad, CA) and 1.25 units *Taq* DNA polymerase. rRNA gene levels in root DNA were quantified by interpolation using a standard curve comprised of a dilution series of cloned rRNA genes.

### Nucleotide Sequence Data

The nucleotide sequences of the following rRNA genes reported in this paper have been deposited in GenBank (JQ973613-JQ973615) and Sequence Read Archives (SRA049976.1).

## Supporting Information

Table S1 **Reverse PCR primers used in the Illumina-based high throughput sequence analysis of bacterial 16S rRNA genes.** Each reverse PCR primer is comprised of the 4 adjoining segments in each row shown above.
(DOCX)

Table S2 **Forward PCR primer used in the Illumina-based high throughput sequence analysis of bacterial 16S rRNA genes.** The forward PCR primer is comprised of the 3 adjoining segments shown above.
(DOCX)

Table S3 **Sequencing primers used in the Illumina-based high throughput sequence analysis of bacterial 16S rRNA genes.**
(DOCX)
