# Supplementary material for: Correlations between Root-Associated Microorganisms and Peach Replant Disease Symptoms in a California Soil
Source: PLoS One. 2012 Oct 5;7(10):e46420. doi: 10.1371/journal.pone.0046420 (PMC3465339; doi:10.1371/journal.pone.0046420)
Supplement: Table S1 — Reverse PCR primers used in the Illumina-based high throughput sequence analysis of bacterial 16S rRNA genes. Each reverse PCR primer is comprised of the 4 adjoining segments in each row shown above. (DOCX) [file pone.0046420.s001.docx]

Table S1. Reverse PCR primers used in the Illumina-based high throughput sequence analysis of bacterial 16S rRNA genes.

| No. | 3’ Illumina Adapter | Barcode | Spacer | Reverse 16S Primer (R806) |
| --- | --- | --- | --- | --- |
| 1 | CAAGCAGAAGACGGCATACGAGAT | CTAGCGTGCGTT | AGTCAGTCAGCC | GGACTACHVGGGTWTCTAAT |
| 2 | CAAGCAGAAGACGGCATACGAGAT | TCGACATCTCTT | AGTCAGTCAGCC | GGACTACHVGGGTWTCTAAT |
| 3 | CAAGCAGAAGACGGCATACGAGAT | ACGAGACTGATT | AGTCAGTCAGCC | GGACTACHVGGGTWTCTAAT |
| 4 | CAAGCAGAAGACGGCATACGAGAT | CGAGTCACGATT | AGTCAGTCAGCC | GGACTACHVGGGTWTCTAAT |
| 5 | CAAGCAGAAGACGGCATACGAGAT | GCCATAGTGTGT | AGTCAGTCAGCC | GGACTACHVGGGTWTCTAAT |
| 6 | CAAGCAGAAGACGGCATACGAGAT | GTAGACATGTGT | AGTCAGTCAGCC | GGACTACHVGGGTWTCTAAT |
| 7 | CAAGCAGAAGACGGCATACGAGAT | TAGACACCGTGT | AGTCAGTCAGCC | GGACTACHVGGGTWTCTAAT |
| 8 | CAAGCAGAAGACGGCATACGAGAT | CGGATCTAGTGT | AGTCAGTCAGCC | GGACTACHVGGGTWTCTAAT |
| 9 | CAAGCAGAAGACGGCATACGAGAT | GACCACTGCTGT | AGTCAGTCAGCC | GGACTACHVGGGTWTCTAAT |
| 10 | CAAGCAGAAGACGGCATACGAGAT | ATGAAGCACTGT | AGTCAGTCAGCC | GGACTACHVGGGTWTCTAAT |
| 11 | CAAGCAGAAGACGGCATACGAGAT | TCGCGCAACTGT | AGTCAGTCAGCC | GGACTACHVGGGTWTCTAAT |
| 12 | CAAGCAGAAGACGGCATACGAGAT | GCTAAGTGATGT | AGTCAGTCAGCC | GGACTACHVGGGTWTCTAAT |
| 13 | CAAGCAGAAGACGGCATACGAGAT | CACGTGACATGT | AGTCAGTCAGCC | GGACTACHVGGGTWTCTAAT |
| 14 | CAAGCAGAAGACGGCATACGAGAT | TGCGCTGAATGT | AGTCAGTCAGCC | GGACTACHVGGGTWTCTAAT |
| 15 | CAAGCAGAAGACGGCATACGAGAT | GATGTATGTGGT | AGTCAGTCAGCC | GGACTACHVGGGTWTCTAAT |
| 16 | CAAGCAGAAGACGGCATACGAGAT | GCATCGTCTGGT | AGTCAGTCAGCC | GGACTACHVGGGTWTCTAAT |
| 17 | CAAGCAGAAGACGGCATACGAGAT | CTAGTCGCTGGT | AGTCAGTCAGCC | GGACTACHVGGGTWTCTAAT |
| 18 | CAAGCAGAAGACGGCATACGAGAT | TCTGATCGAGGT | AGTCAGTCAGCC | GGACTACHVGGGTWTCTAAT |
| 19 | CAAGCAGAAGACGGCATACGAGAT | GATAGCACTCGT | AGTCAGTCAGCC | GGACTACHVGGGTWTCTAAT |
| 20 | CAAGCAGAAGACGGCATACGAGAT | TGGTCGCATCGT | AGTCAGTCAGCC | GGACTACHVGGGTWTCTAAT |
| 21 | CAAGCAGAAGACGGCATACGAGAT | TAGCAGTTGCGT | AGTCAGTCAGCC | GGACTACHVGGGTWTCTAAT |
| 22 | CAAGCAGAAGACGGCATACGAGAT | GTATCTGCGCGT | AGTCAGTCAGCC | GGACTACHVGGGTWTCTAAT |
| 23 | CAAGCAGAAGACGGCATACGAGAT | TCCAGATAGCGT | AGTCAGTCAGCC | GGACTACHVGGGTWTCTAAT |
| 24 | CAAGCAGAAGACGGCATACGAGAT | GACACTCACCGT | AGTCAGTCAGCC | GGACTACHVGGGTWTCTAAT |
| 25 | CAAGCAGAAGACGGCATACGAGAT | TGCTACAGACGT | AGTCAGTCAGCC | GGACTACHVGGGTWTCTAAT |
| 26 | CAAGCAGAAGACGGCATACGAGAT | TCTACGGCACGT | AGTCAGTCAGCC | GGACTACHVGGGTWTCTAAT |
| 27 | CAAGCAGAAGACGGCATACGAGAT | GTGTGCTAACGT | AGTCAGTCAGCC | GGACTACHVGGGTWTCTAAT |
| 28 | CAAGCAGAAGACGGCATACGAGAT | ATAGGCTGTAGT | AGTCAGTCAGCC | GGACTACHVGGGTWTCTAAT |
| 29 | CAAGCAGAAGACGGCATACGAGAT | ACCACACGTAGT | AGTCAGTCAGCC | GGACTACHVGGGTWTCTAAT |
| 30 | CAAGCAGAAGACGGCATACGAGAT | TATGGAGCTAGT | AGTCAGTCAGCC | GGACTACHVGGGTWTCTAAT |
| 31 | CAAGCAGAAGACGGCATACGAGAT | GAGTATCTGAGT | AGTCAGTCAGCC | GGACTACHVGGGTWTCTAAT |
| 32 | CAAGCAGAAGACGGCATACGAGAT | ATCGAATCGAGT | AGTCAGTCAGCC | GGACTACHVGGGTWTCTAAT |

Each reverse PCR primer is comprised of the 4 adjoining segments in each row shown above.
