# Supplementary material for: Correlations between Root-Associated Microorganisms and Peach Replant Disease Symptoms in a California Soil
Source: PLoS One. 2012 Oct 5;7(10):e46420. doi: 10.1371/journal.pone.0046420 (PMC3465339; doi:10.1371/journal.pone.0046420)
Supplement: Table S2 — Forward PCR primer used in the Illumina-based high throughput sequence analysis of bacterial 16S rRNA genes. The forward PCR primer is comprised of the 3 adjoining segments shown above. (DOCX) [file pone.0046420.s002.docx]

Table S2. Forward PCR primer used in the Illumina-based high throughput sequence analysis of bacterial 16S rRNA genes.

| Name | 5’ Illumina Adapter | Spacer | Forward 16S Primer (F515) |
| --- | --- | --- | --- |
| IL-SSU-PCR-F | AATGATACGGCGACCACCGAGATCTACAC | TATCGCCGTTGT | GTGCCAGCMGCCGCGGTAA |

The forward PCR primer is comprised of the 3 adjoining segments shown above.
