# Supplementary material for: Correlations between Root-Associated Microorganisms and Peach Replant Disease Symptoms in a California Soil
Source: PLoS One. 2012 Oct 5;7(10):e46420. doi: 10.1371/journal.pone.0046420 (PMC3465339; doi:10.1371/journal.pone.0046420)
Supplement: Table S3 — Sequencing primers used in the Illumina-based high throughput sequence analysis of bacterial 16S rRNA genes. (DOCX) [file pone.0046420.s003.docx]

Table S3. Sequencing primers used in the Illumina-based high throughput sequence analysis of bacterial 16S rRNA genes.

| Name | Purpose | Sequence |
| --- | --- | --- |
| IL-SSU-Read-1 | 5’ sequencing primer | TATCGCCGTTGTGTGCCAGCMGCCGCGGTAA |
| IL-SSU-Index | Barcode sequencing primer | ATTAGAWACCCBDGTAGTCCGGCTGACTGACT |
